# Water Quality Impact and Nature-Based Solutions Guidelines Caused by Sanitary Landfill Site

**DOI:** 10.21315/tlsr2026.37.3.12

**Published:** 2026-10-15

**Authors:** Natthapong Iadtem, Phongthon Saengchut, Siraphob Obpat, Pattrawadee Klaiklung, Matura Angkaew, Chuti Rakasachat

**Affiliations:** 1 Department of Environmental Technology and Management, Faculty of Environment, Kasetsart University, Ngamwongwan Rd., Lat Yao, Chatuchak, Bangkok 10900, Thailand; 2 Department of Biological and Health Science, Faculty of Science, Ubon Ratchathani University, 85 Sathonlamark Road, Mueang Si Khai, Ubon Ratchathani 34190, Thailand; 3 Center of Research and Academic Services, Faculty of Environment, Kasetsart University, Ngamwongwan Rd., Lat Yao, Chatuchak, Bangkok 10900, Thailand; 4 Department of Environment for Sustainability, Faculty of Environment, Kasetsart University, Ngamwongwan Rd., Lat Yao, Chatuchak, Bangkok 10900, Thailand

**Keywords:** Environmentally Friendly, Sanitary Landfill, Sustainable Management, Wastewater, Water Quality

## Abstract

This study to monitor and assess the environmental impact of a sanitary landfill in Chachoengsao Province on surrounding water resources between 2023 and 2025 including usage guidelines of nature-based solutions (NBS) for wastewater management. Waste traps and monitoring ponds consistently showed extremely high water quality index values, with monthly averages reaching up to 240,000, which Thailand classifies as inferior water quality index > 100. This severe pollution was primarily due to excessive concentrations of biochemical oxygen demand-5 > 23,000 milligrams per liter, ammonia-nitrogen > 1,000 milligrams per liter and Total coliform bacteria > 160,000 most probable number/100 milliliter. In contrast, surface water displayed moderate to good water quality index (28–40), while groundwater from deep wells generally maintained good water quality index (25–30), with no detection of heavy metals exceeding safety standards. The multivariate statistical analyses using principal component analysis a principal coordinate analysis identified biochemical oxygen demand-5, ammonia-nitrogen, total coliform bacteria and fecal coliform bacteria as key indicators, influenced by both seasonal factors and pollution sources. Water quality around the landfill site showed differences in pollution levels, especially in the waste trap and monitoring pond, and very high water quality index values at various times, which are classified as “inferior water quality”. Surface water sources had water quality in the good to moderate range and the groundwater from deep wells remained of good quality with no heavy metals or toxic substances detected at levels exceeding the standard.

**HIGHLIGHTS**

- Landfill leachate caused severe water quality deterioration at source points.
- PCA and PCoA identified seasonal patterns of water contamination.
- Nature-based solutions offer sustainable landfill leachate management.

## INTRODUCTION

Solid waste management remains a serious global environmental challenge, particularly in developing countries, where sanitary landfilling is the predominant method for disposing of municipal solid waste (MSW) (Hussein *et al*. 2021; Peterside *et al*. 2022; Naz *et al*. 2024). Sanitary landfills incorporate engineered controls, including leachate containment systems, landfill liners, gas collection systems and daily cover applications, to prevent uncontrolled releases of contaminants into the environment. However, even landfills designed with advanced technologies can pose substantial long-term environmental risks when maintenance is inadequate or monitoring is insufficient (Hussein *et al*. 2021; Hale *et al*. 2022).

A major concern is the generation and migration of leachate, a toxic liquid formed when precipitation percolates through waste, producing a complex mixture of organic pollutants, ammonium, heavy metals (e.g., lead, cadmium and mercury) and other potentially hazardous substances. If not adequately controlled, leachate can migrate through liner defects or preferential flow paths (e.g., fracture zones) and eventually enter groundwater, causing contamination. Numerous studies have reported that leachate from poorly managed or malfunctioning landfill sites can reach groundwater (Peterside *et al*. 2022; Mor *et al*. 2023; Naz *et al*. 2024).

Nature-based solutions (NBS) have received increasing attention as sustainable approaches to environmental pollution control, particularly for treating leachate from MSW disposal sites (Hale *et al*. 2023; Zalaznik *et al*. 2023). Leachate, a highly polluted liquid generated during waste decomposition, contains elevated concentrations of organic matter, heavy metals, excess nutrients and persistent toxic substances. If left untreated, it can severely affect surface water, groundwater and surrounding ecosystems. NBS, such as constructed wetlands multistage bioponds and vegetated buffer zones, provide environmentally friendly treatment by harnessing natural processes involving aquatic plants, soils and microorganisms to degrade, transform or immobilise contaminants. These systems can reduce reliance on conventional treatment technologies while enhancing biodiversity and improving green landscapes at waste disposal sites (Aoife *et al*. 2024; Vassalle *et al*. 2023; Mohd Nazir *et al*. 2025).

Leachate composition varies with landfill age. Young landfills (< 5 years old) typically generate leachate with low pH and high biochemical oxygen demand-5 (BOD_5_) and chemical oxygen demand (COD), with relatively high BOD_5_/COD ratios and moderate ammonia concentrations, indicating substantial organic and inorganic pollution. In contrast, old landfills (> 10 years old) generally produce more stable leachate characterised by high ammonia concentrations, moderate BOD_5_ and COD levels, and lower BOD_5_/COD ratios. Leachate generation can persist for many years after landfill closure. These changes can be early indicators of groundwater contamination and underscore the need for routine and strategically designed groundwater monitoring at landfill sites (Iwar *et al*. 2021; Peterside *et al*. 2022; Naz *et al*. 2024).

In some countries, regulations require monitoring of specific parameters near landfills; however, evidence suggests that these minimum requirements may not capture the full extent of environmental impacts. Therefore, modern assessment tools such as the water quality index (WQI) are widely used to assess suitability for human use and ecological function by integrating multiple physical, chemical and biological parameters into a single composite value. This approach simplifies complex datasets, supports clearer communication of water quality status to policymakers, researchers and the public, and enables comparison across locations and time periods (Iwar *et al*. 2021; Peterside *et al*. 2022; Mor *et al*. 2023; Naz *et al*. 2024).

Chachoengsao Province, located in eastern Thailand, is undergoing rapid industrial development and urban expansion. As waste generation increases, sanitary landfills have remained the primary disposal method. However, some landfill sites are located near groundwater-dependent communities and agricultural areas, raising concerns about long-term leachate infiltration and deterioration of water quality. Through systematic sampling and analysis of physical and chemical groundwater parameters near a landfill site, this study aimed to identify changes in water quality and evaluate pollution levels using contamination indices. The findings are expected to inform sustainable waste management policies, strengthen environmental monitoring strategies, and provide a scientific basis for mitigation and remediation planning in comparable landfill-impacted settings. Accordingly, this study aimed to monitor and evaluate the impacts of a sanitary landfill in Chachoengsao Province on surrounding water resources.

## MATERIALS AND METHODS

### Sampling and Experimental Analysis

The study area comprised a sanitary landfill site in Chachoengsao Province (Fig.1). Water samples were collected four times per year (January, April, July and November) from 2023 to 2025. To capture spatial and temporal variability, the study employed individual grab sampling at three randomly selected replicate points within each of the four designated location types: garbage traps, surface water (Khlong Tha Lat and Huai Ban Song), groundwater and monitoring wells (pond 1, pond 2 and pond 3). Sampling points were strategically located within and around the landfill area to represent the surrounding environmental conditions and capture potential gradients of contamination. This randomised sampling approach was used to identify episodic fluctuations in water quality and leachate concentrations, which can vary significantly due to seasonal factors and rainfall events. This design yielded 12 samples per sampling campaign, totaling 48 samples per year for comprehensive laboratory characterisation. Samples were collected in sterile 1.5 L polyethylene bottles and transported to the laboratory in an ice chest at 4°C.

*In situ* water properties, including potential of hydrogen (pH), dissolved oxygen (DO) and temperature, were measured using electrometric methods. Turbidity was determined using a nephelometric method. Total hardness was determined by titration with ethylenediaminetetraacetic acid (EDTA). Total solids (TS) were determined by drying at 103°C–105°C. Colour was measured using a test kit. Ammonia-nitrogen (NH_3_-N) was determined by distillation, and nitrate-nitrogen (NO^−^_3_ -N) was determined using the cadmium reduction method. Total coliform bacteria (TCB) and fecal coliform bacteria (FCB) were determined using the most probable number (MPN) test. Biochemical oxygen demand (BOD_5_) was determined using azide modification (20°C, 5 days), and chemical oxygen demand (COD) was determined using closed reflux. *Escherichia coli* (*E. coli*) and standard plate count (SPC) were determined using colony count methods. Cyanide (CN) was determined using distillation and colorimetric methods. Manganese (Mn) was determined using persulfate. Fluoride (F^−^) was determined using a colorimetric method. Chloride (Cl^−^) was determined using a silver nitrate (AgNO_3_) titrimetric method. Sulfate (SO^2−^_4_) was determined using turbidimetric method. Arsenic (As), cadmium (Cd), Iron (Fe), hexavalent chromium (Cr^6+^), copper (Cu), lead (Pb), mercury (Hg), nickel (Ni), selenium (Se) and zinc (Zn) were determined using an atomic absorption spectrophotometer (AAS) (Nguyen *et al*. 2023; Tosin & Okoro 2023; Ranpura *et al*. 2025).

### Water Quality Index (WQI)

Water quality index (WQI) is a widely accepted method for assessing the suitability of water for human use and ecological function by integrating multiple physical, chemical and biological parameters into a single composite value, which supports the selection of treatment techniques (Ilaboya *et al*. 2014; Luo *et al*. 2024; Swami & Tyagi 2000; 2007). This approach simplifies complex water quality data, enables more effective communication of water quality status to policymakers, researchers and the public, and allows comparisons across different locations or time periods (Shakarami *et al*. 2025; Tyagi *et al*. 2013). In practice, each parameter is assigned a weight (w_i_) based on its relative importance to overall water quality, typically guided by standards. The weights are used to calculate the relative weight (W_i_) and the quality rating scale (q_i_), which are then combined to obtain the final WQI. Parameters with greater impacts on health and water availability were assigned higher weights, reflecting their key role in determining water quality (Vasanthavigar *et al*. 2010; Srinivasamoorthy *et al*. 2008; Singh *et al*. 2016; Kawo & Karuppannan 2018; Nayar 2020).

In this study, WQI was used as a composite indicator to assess water quality at multiple sampling locations around the sanitary landfill. The WQI calculation incorporated multiple parameters, including pH, BOD_5_, NO^−^_3_-N, TCB, chloride (Cl^−^) and total hardness, depending on data availability and relevance to each water type. To ensure an appropriate contribution of each parameter, a weight value (w_i_) was assigned based on its relative significance to environmental and public health. These weights were normalised to derive the relative weight (W_i_) using W_i_ = w_i_ / ∑w_i_ (Peterside *et al*. 2022; Naz *et al*. 2024).


(1) $$\text{Wi}=\frac{\text{Wi}}{\sum_{\text{i}=1}^{\text{n}}\text{Wi}}$$

Each parameter’s quality rating (q_i_) was calculated using Equation (2) (Peterside *et al*. 2022; Naz *et al*. 2024):


(2) $$\text{qi}=\frac{\text{Ci}}{\text{Si}}\times 100$$

Where; C_i_ is the measured concentration and S_i_ is the standard permissible limit. The sub-index (SI_i_) for each parameter was then calculated using Equation (3) (Peterside *et al*. 2022; Naz *et al*. 2024):


(3) $${\text{SI}}_{\text{i}}={\text{q}}_{\text{i}}\times {\text{W}}_{\text{i}}$$

The overall WQI value was obtained by summing all SI_i_ values using Equation (4) (Peterside *et al*. 2022; Naz *et al*. 2024):


(4) $$\text{WQI}=\sum_{\text{i}=1}^{\text{n}}{\text{SI}}_{\text{i}}$$

WQI is the water quality index, the range of water quality rating is presented in Table 1.

### Principal Component Analysis (PCA) and Principal Coordinate Analysis (PCoA)

PCA and PCoA are dimensionality reduction techniques. Separate analyses were performed for heavy metals and for physico-chemical parameters. Variables were grouped based on similar scales and variability, which may reveal influences on groundwater, garbage traps, monitoring wells and surface water (Mukherjee *et al*. 2022; Chawishborwornworng *et al*. 2024). PCA was used to identify potential sources of heavy metals and physico-chemical variables. PCA reduces data dimensionality (Manikandan *et al*. 2014) and supports identification of pollution sources (Gibrilla *et al*. 2011). Varimax rotation with Kaiser normalisation was applied for factor analysis (Howitt & Cramer 2005) to facilitate interpretation by minimising the number of variables with high loadings on each component (Boateng *et al*. 2015). PCoA was performed using Bray–Curtis dissimilarity (Bai *et al*. 2018) and can accommodate a wider range of distance or similarity measures, making it suitable for various data types, including qualitative or discrete variables (Morrissey *et al*. 2017).

## RESULTS

### Water Quality Characteristics

Water quality evaluation at the garbage trap sampling points (Table 2) indicated that pH ranged from 4.70 to 8.90, with a mean of 6.97 ± 1.62. BOD_5_ ranged from 1,566.30 mg/L to 23,500.00 mg/L (mean 9,550.32 ± 9,790.76 mg/L), indicating very high organic loading above permissible limits. NH_3_-N ranged from 45.10 to 1,078.20 mg/L (mean 317.12 ± 485.80 mg/L), suggesting persistent pollution consistent with inputs of untreated sewage. NO^−^_3_-N was reported to vary from 45.10 mg/L to 1,078.20 mg/L, with a mean of 36.98 ± 58.56 mg/L, potentially associated with fertiliser inputs and/or nitrification of NH_4_^+^ under more aerobic conditions. TCB and FCB were consistently > 160,000 MPN/100 mL, indicating a high risk of waterborne pathogens. Temperatures ranged from 24.70°C to 26.20°C (mean 25.47 ± 0.63°C), indicating minimal spatial and temporal variation. COD ranged from 2,984.00 mg/L to 29,800.00 mg/L (mean 15,940.17 ± 13,516.58 mg/L), consistent with the elevated BOD_5_ and low DO. CN ranged from 0.09 mg/L to 0.25 mg/L (mean 0.19 ± 0.06 mg/L), suggesting possible contamination associated with industrial chemical waste. Heavy metal concentrations were reported as follows: As, 0.21–0.42 mg/L (mean 0.57 ± 0.73 mg/L); Cd, 0.17–0.84 mg/L (mean 0.54 ± 0.26 mg/L); Cr^6+^, 0.30–2.17 mg/L (mean 1.48 ± 0.62 mg/L); Cu, 0.25–0.91 mg/L (mean 0.07 ± 0.21 mg/L); Mn, 0.32–60.67 mg/L (mean 9.47 ± 19.42 mg/L); Pb, 0.35–0.80 mg/L (mean 0.64 ± 0.14 mg/L); Hg, 0.12–0.66 mg/L (mean 0.40 ± 0.16 mg/L); Ni, 0.19–0.77 mg/L (mean 0.46 ± 0.21 mg/L); and Zn, 1.50–3.02 mg/L (mean 2.48 ± 0.48 mg/L).

Surface water assessment (Table 2) indicated pH values of 6.90–8.00 (mean 7.62 ± 0.35). DO ranged from 5.30 to 8.70 mg/L (mean 6.88 ± 1.15 mg/L), which exceeded the minimum guideline value (≥ 2.00 mg/L) and indicated good oxygenation, potentially due to surface aeration and relatively low organic inputs. BOD_5_ ranged from 0.30 mg/L to 2.00 mg/L (mean 1.07 ± 0.55 mg/L), remaining below the standard threshold (< 4.00 mg/L). NH_3_-N ranged from 0.25 to 1.00 mg/L (mean 0.41 ± 0.23 mg/L) and remained below 0.50 mg/L in most samples. NO^−^_3_-N ranged from 0.07 mg/L to 1.29 mg/L (mean 0.58 ± 0.64 mg/L), consistently below 5.00 mg/L. TCB ranged from 15.00 to 240.00 MPN/100 mL (mean 86.67 ± 58.23 MPN/100 mL), remaining below 20,000 MPN/100 mL, while FCB was consistently < 1.80 MPN/100 mL, well below 4,000.00 MPN/100 mL. Temperature ranged from 24.70°C to 26.30°C (mean 25.32 ± 0.64 °C), which was typical for surface waters. COD remained <40.00 mg/L, consistent with clean to moderately impacted conditions. Mn occurred at very low concentrations (< 0.53 mg/L) and was typically below detection or well under the standard (< 1.00 mg/L). Heavy metals (As, Cd, Cr^6+^, Cu, Pb, Hg, Ni and Zn) and CN were not detected (N.D.). Overall, Khlong Tha Lat and Huai Ban Song maintained stable and acceptable water quality during the monitoring period. However, slight increases in NH_3_-N and NO^−^_3_-N in certain months support continued monitoring, particularly during the rainy season.

Groundwater assessment (Table 2) indicated that overall conditions remained within acceptable limits during the monitoring period. pH ranged from 7.20 to 8.40 (mean 7.76 ± 0.41), within the standard range. BOD_5_ ranged from 0.10 to 4.00 mg/L (mean 1.76 ± 1.57 mg/L), indicating limited organic pollution. TS showed seasonal variation (47.00–211.00 mg/L; mean 85.78 ± 50.33 mg/L). NO^−^_3_-N ranged from 0.03 to 3.11 mg/L (mean 0.41 ± 1.01 mg/L), below 45.00 mg/L. Standard plate counts ranged from 0 to 120.00 CFU/mL (mean 36.88 ± 41.04 CFU/mL), below the permissible limit of 500 CFU/mL. TCB remained < 1.10 MPN/100 mL, and *E. coli* was not detected in any sample. Turbidity remained low (0.01–0.40 NTU; mean 0.09 ± 0.13 NTU), and colour was < 5.00 Hazen, indicating clear and visually acceptable water. COD remained < 40.00 mg/L. Sulfide concentrations were low (mostly < 0.02 mg/L), indicating minimal anaerobic conditions and/or limited sulfide mineral dissolution in the aquifer. Fe, F, Mn, SO4 and Cl^−^ were detected at trace concentrations well below standard limits. Total hardness ranged from 1.95 mg/L to 77.91 mg/L (mean 21.62 ± 22.55 mg/L), classifying the groundwater as soft. Heavy metals (As, Cd, CN, Pb, Hg, Se and Zn) were not detected (N.D.). Collectively, these results indicated that groundwater remained safe, with no evidence of pollution or degradation during the three-year monitoring period.

Monitoring well water quality at three sampling points (pond 1, pond 2 and pond 3) (Table 2) indicated stable and generally acceptable conditions. pH ranged from 6.80 to 8.30 (mean 7.49 ± 0.46), indicating neutral to slightly alkaline water. BOD_5_ ranged from 4.00 to 13.00 mg/L (mean 6.84 ± 4.29 mg/L); higher values observed in July 2023 suggested episodic organic inputs, although values remained within permissible limits. DO ranged from 5.70 to 8.60 mg/L (mean 6.71 ± 1.05 mg/L), sufficient to support aerobic processes. NH_3_-N varied from 1.00 to 8.00 mg/L (mean 5.19 ± 1.67 mg/L), with a maximum of 7.05 mg/L (pond 2) in July 2023, suggesting episodic inputs and/or infiltration of organic matter. NO^−^_3_-N ranged from 0.08 to 2.32 mg/L (mean 0.61 ± 0.83 mg/L). TCB and FCB were largely undetectable (< 1.80 MPN/100 mL), indicating minimal fecal contamination; however, an elevated TCB value in January 2023 from pond 1 (95.00 MPN/100 mL) warrants investigation of potential contamination sources during that period. Temperature ranged from 25.50°C to 28.50°C (mean 25.36 ± 0.63°C), reflecting seasonal conditions. COD ranged from 40.00 mg/L to 73.00 mg/L (mean 51.09 ± 11.08 mg/L), suggesting limited variability in oxidisable constituents. Heavy metals (As, Cd, Cr^6+^, Pb, Hg, Ni, Cu, Mn and Zn) were consistently not detected (N.D.). Overall, water quality across the three monitoring wells remained in good condition and generally compliant with environmental standards.

### Evaluation of Water Quality Based on WQI

The WQI framework interprets higher index values as indicating higher pollution levels, particularly when pollutants such as BOD_5_ or coliform bacteria exceed guideline thresholds. Conversely, lower WQI values indicate cleaner and safer water with lower pollutant levels, suggesting minimal environmental and public health risks. Therefore, WQI has an inverse relationship with water quality, which is a fundamental characteristic of most water quality index models. This approach facilitates interpretation of complex water quality datasets by integrating multiple physico-chemical and microbiological parameters into a single index value (Table 3).

In this study, groundwater samples were assessed using Thailand’s groundwater quality standards, issued by the Department of Groundwater Resources. Water samples from the garbage trap, surface water, and monitoring wells were assessed using the Surface Water Quality Standards (Type 3) issued by the Pollution Control Department (PCD) of Thailand, which are suitable for agricultural use or industrial water sources requiring treatment (Pollution Control Department 2020). Sampling was conducted in January, April, July and November.

The WQI results indicated distinct gradations of pollution and environmental risk among the different water sources. The garbage trap consistently exhibited the highest WQI values, often exceeding 1,000 under the classification system, indicating very poor water quality and suggesting that the water was highly polluted and unsuitable for use without extensive treatment. Elevated WQI values were primarily driven by high BOD_5_ and total coliform bacteria levels, indicating severe organic and microbial contamination. These findings are consistent with previous landfill studies reporting that infiltration and runoff contribute to the degradation of surface water quality (Peterside *et al*. 2022; Rahmanian *et al*. 2015). It should be noted that the exceptionally high WQI values observed in this study (exceeding 200,000 in some cases) are a direct consequence of the calculation approach combined with the extremely elevated pollutant concentrations in landfill leachate. The WQI was computed using a weighted aggregation method, in which each parameter was transformed into a sub-index and multiplied by its respective weighting factors. When parameter concentrations, particularly BOD_5_ and microbial indicators, greatly exceed standard guideline values by several orders of magnitude, their corresponding sub-index values increase disproportionately, resulting in very large final WQI scores. Unlike typical surface water assessments, landfill leachate is characterised by extremely high organic loads (e.g., very high BOD_5_) and microbial contamination, which can exceed regulatory limits by tens to hundreds of times over. Under such conditions, the applied WQI formulation does not impose an upper cap on sub-index values and therefore produces amplified scores that reflect the severity of pollution rather than remaining within conventional WQI ranges (e.g., < 1,000). Therefore, the reported WQI values should be interpreted as indicative of extreme pollution intensity rather than being directly comparable to the typical WQI classifications used for natural surface waters. This approach allows for a clearer differentiation of highly contaminated sites, particularly those impacted by landfills. Surface water stations evaluated using PCD Type 3 standards showed seasonal fluctuations. During the wet season in Thailand, precipitation can increase pollutant loads, including suspended solids, metals, and microbes (Peterside *et al*. 2022). Extremely high WQI values are uncommon in natural surface water systems and may reflect episodic influxes of pollution, including leachate-related components from landfills (Naz *et al*. 2024). Comparable seasonal patterns have been reported in studies from Southeast Asia and West Africa, where the river WQI increased substantially during monsoon periods (Rahmanian *et al*. 2015). Monitoring wells frequently exhibited WQI values exceeding 100, placing them in the “poor” to “very poor” category. Under standard WQI classifications, these values indicate potential contamination concerns and may reflect limitations in landfill lining performance and/or leachate containment effectiveness of the landfill. In contrast, deeper groundwater samples showed WQI values ranging from 25 to 50, corresponding to “good to moderate” quality water. These relatively low WQI values suggest that natural attenuation processes have reduced contaminant mobility and concentrations, consistent with studies reporting lower WQI scores in confined or better-protected groundwater zones, even when located near contaminated sites (Peterside *et al*. 2022).

Overall, the WQI framework supported the identification of high-risk zones, particularly at the garbage trap and monitoring wells, where values frequently exceeded 100 and indicated unacceptable conditions. The WQI also supports evidence-based decision-making by translating complex water chemistry into a format that is readily interpretable by scientists, decision-makers and stakeholders, including policymakers, community members and municipal authorities. This is particularly relevant when pollutants such as BOD_5_, nitrate, or coliform bacteria occur at concentrations above the permissible thresholds, resulting in elevated sub-indices and overall WQI values. Lower WQI values indicate lower pollutant burdens and correspondingly lower environmental and public health risks. This inverse relationship between WQI and water quality has been a defining feature of weighted arithmetic WQI models, including the model applied in this study (Naz *et al*. 2024; Uddin *et al*. 2022). Accordingly, the WQI has facilitated the visual ranking of water bodies by pollution severity and has supported the prioritisation of monitoring, regulatory enforcement and remediation in critical zones (Islam & Rahman 2023; Tyagi *et al*. 2013). Wongaree (2019) assessed the Mak Khaeng Canal in Udon Thani as having “poor” water quality according to the PCD-WQI, which was attributed to untreated wastewater discharges from the Udon Thani Municipality. Key drivers of pollution include rapid economic growth, urbanisation and anthropogenic activities, with elevated BOD5, FCB and TCB linked to densely populated communities along canals. This situation is exacerbated by inadequate sewage infrastructure and uncontrolled wastewater disposal from households, educational institutions, offices, agricultural activities and commercial operations. These findings highlight priority areas for improvement and provide guidance for policymakers in developing water management strategies that incorporate collaboration with local communities. The analysis (Fig. 2) indicates that across all months, two sites (garbage trap and monitoring well) remained in the “very poor” category, whereas surface water and groundwater generally exhibited good water quality. The WQI results suggest that the surface water and groundwater in the study area are suitable for drinking purposes, although some locations are not suitable for drinking (Gautam *et al*. 2024; Tyagi *et al*. 2013).

### Evaluation of Water Quality Based on Principal Components Analysis (PCA)

The implementation of PCA and PCoA served as critical diagnostic tools for deciphering the complex hydrochemical and microbiological interactions across landfill-impacted environments. By transforming a high-dimensional dataset of physicochemical and biological variables into a set of orthogonal Principal Components (PCs), this study effectively isolated the primary drivers of water quality degradation and identified the spatiotemporal signatures of contamination.

The multivariate assessment of the garbage trap samples (Fig. 3) illustrated the extreme chemical and microbial complexity of raw leachate. In the PCA biplot (Fig. 3a), the primary axis (PC1) was predominantly defined by heavy metals (Zn, Cu, Cr^6+^, Hg and Pb) and organic indicators (BOD_5_ and COD). The strong positive loading of these metals on PC1 indicates a common source related to the dissolution of electronic and industrial waste within the landfill. PCoA (Fig. 3b) further differentiated the samples based on seasonal pulses. The clustering of early year samples (e.g., 2023_January) along the vectors for NH_3_-N, NO^−^_3_-N, and as suggests that during periods of low dilution, leachate is characterised by high nitrogenous loads and toxic metalloids. Conversely, the negative loading of pH relative to the organic vectors confirms that as decomposition intensifies (indicated by a higher BOD_5_/COD), the environment becomes more acidic, further facilitating the mobility of heavy metals into the surrounding ecosystem.

The analysis of surface water (Fig. 4) reveals a distinct “microbial-nutrient gradient.” In the PCA biplot (Fig. 4a), the vectors for TCB and FCB exhibited high positive loadings on PC2, whereas BOD_5_ and COD dominated the negative PC1 space. This structure suggests that surface water quality is governed not only by organic loading but also by microbial contamination from urban runoff. PCoA (Fig. 4b) revealed spatial differentiation between the two rivers. Samples from Khlong Tha Lat (PKh) tended to align more closely with the TCB and FCB vectors than those from Huai Ban Song (PHu), indicating a higher degree of anthropogenic fecal pollution in the former. Furthermore, the opposing directions of the DO and NH_3_-N vectors statistically validate the oxygen-depleting nature of the ammonia-rich wastewater discharge. These findings align with those of Luo *et al*. (2024), who noted that PCA can effectively distinguish between natural background variations and localised anthropogenic microbial spikes in riverine systems.

The PCA for the monitoring wells (P1, P2 and P3) (Fig. 5) served as an early warning indicator for subsurface leachate migration. The loading vectors for TCB, BOD_5_, and Mn were tightly clustered and showed high negative loadings on PC1 (Fig. 5a), suggesting a strong inter-correlation between microbial presence and organic-driven redox changes. Specifically, the high magnitude of the Mn vector in the same direction as BOD_5_ suggests that organic matter infiltration from the landfill triggers the reductive dissolution of Mn-bearing minerals in the aquifer. The PCoA (Fig. 5b) demonstrated that samples from Pond 1 (P1) and Pond 2 (P2) often shifted toward these contamination vectors during the early months of the year (January and April). This seasonal “clustering” suggests that rainfall-induced infiltration facilitates the transport of both organic matter and bacteria into the shallow groundwater system, as observed by Nadaraja *et al*. (2017) regarding aquifer recharge dynamics.

In the groundwater dataset (Fig. 6), the PCA biplot (Fig. 6a) identified a primary axis governed by mineralisation and turbidity. The positive loadings of Cl^−^, Total Hardness and Color on PC1 reflected the inherent hydrogeochemical properties of the aquifer (mineral dissolution). However, the presence of Standard Plate Count (SPC) and BOD_5_ vectors in the PC1 positive space indicate that the deep aquifer is periodically susceptible to external infiltration. The PCoA (Fig. 6b) revealed a clear separation: while many samples clustered around the stable pH and SO^2−^_4_ vectors, several early year samples (e.g., 2023_January) were pulled toward the TS and microbial vectors. This suggests that while deep groundwater generally maintains high quality, “preferential flow paths” may exist that allow for rapid seasonal microbial pulses from the surface to the deep aquifer, a critical finding for long-term water safety management.

Overall, the PCA results consistently identified BOD_5_, NH_3_-N, TCB and FCB as the most influential variables across all four water environments. The stability of these vectors across different matrices confirms their reliability as markers of landfill impact. Statistically, the NBS recommended in this study are justified because they specifically target the high-loading organic and nutrient vectors identified in the PCA. By addressing the primary sources of variance (organic and microbial loading), NBS can mitigate contamination gradients before they escalate into irreversible regional water quality degradation.

### Nature-Based Solutions (NBS) Guidelines

NBS are recommended as a relevant approach for mitigating pollution associated with leachate from a municipal solid waste landfill site in Chachoengsao Province. This section addresses contamination driven by leachate discharge generated during municipal solid waste degradation. Leachate typically contains persistent organic compounds, inorganic pollutants such as NH_3_-N and NO_3_^−^-N, heavy metals including Pb, Cd, and Hg, and pathogenic microorganisms. In this study, BOD_5_, NH_3_-N and TCB concentrations in the leachate collection pond and at some monitoring-well locations surrounding the landfill site were very high (more than 23,000 mg/L, over 1,000 mg/L and above 160,000 MPN/100 mL, respectively), indicating a high pollution risk and potential contaminant transport to adjacent surface water and groundwater. These findings indicate an urgent need for preventive and restorative interventions, for which NBS are recommended as a site-appropriate approach.

NBS comprises environmental management strategies that rely on natural processes. Examples include constructed wetlands, which are recommended for removing excess nutrients and heavy metals through plant uptake and microbially mediated processes; multistage bioponds, which may be designed to treat leachate under controlled conditions via natural biodegradation; and vegetated buffer strips established around the site to reduce contaminant transport to nearby watercourses. Compared with conventional engineering-based treatment systems, NBS may support long-term application with lower costs, reduced environmental impacts and co-benefits such as biodiversity enhancement. In addition, NBS may contribute to transforming landfill sites into green spaces and educational ecological environments.

Water quality at the garbage trap, which functions as a collection point for leachate prior to discharge, showed highly polluted conditions, with BOD_5_ exceeding 23,000 mg/L, NH_3_-N above 1,000 mg/L and coliform bacteria counts over 160,000 MPN/100 mL. These concentrations indicate severe pollution and mean the water is not suitable for discharge without treatment. Therefore, NBS such as constructed wetlands, multistage bioponds and vegetated buffer zones are recommended as potential treatment options for reducing pollutant concentrations before discharge.

The management of municipal solid waste through sanitary landfilling presents substantial environmental challenges, particularly regarding leachate-induced water contamination. To mitigate these impacts, NBS are recommended as a sustainable and cost-effective remediation strategy. By adopting the conceptual framework proposed by Mohd Nazir *et al*. (2025), which delineates NBS through seven fundamental elements—natural processes, sustainability and resilience, biodiversity enhancement, diversity of use, community engagement, cost-effectiveness and engineering design—landfill management may transition toward ecological rehabilitation. Previous studies have reported that NBS interventions, such as constructed wetlands and multistage bioponds, can achieve pollutant removal efficiencies of 80%–95% for BOD_5_ and 70%–90% for NH_3_-N (Hale *et al*. 2023; Noor *et al*. 2025; Vassalle *et al*. 2023).

In the Chachoengsao landfill context, applying these processes at the garbage trap is recommended to reduce pollutant concentrations toward levels comparable to local surface water bodies such as Khlong Tha Lat and Huai Ban Song, with BOD_5_ < 2 mg/L, NH_3_-N < 1 mg/L and microbial contamination within acceptable limits for agricultural or industrial use. In addition, because some monitoring wells showed WQI values > 100–400, NBS implemented as leachate pretreatment may reduce pollutant loads before subsurface infiltration. This would reduce pressure on natural filtering layers and help prevent gradual contaminant release to aquifers. Measures that reduce water–pollutant contact, limit particle dispersion and support remediation of contaminated water and soil are recommended for on-site implementation and may contribute to incremental environmental improvement. NBS also offer opportunities for integrated water resource management at landscape or basin scales by supporting balanced economic, environmental and social outcomes, particularly in watersheds spanning multiple administrative boundaries and involving diverse stakeholders (Zalaznik *et al*. 2023).

## DISCUSSION

The astronomical magnitude of the WQI values observed in the garbage traps—reaching a peak of 240,718.00—reflects the severe intensity of raw, undiluted leachate prior to any environmental attenuation. These figures are a direct result of the weighted aggregation method, where pollutant concentrations such as and TCB exceed the Thailand PCD Type 3 Surface Water Standards by several orders of magnitude. As noted by Peterside *et al*. (2022) and Naz *et al*. (2024), the weighted arithmetic WQI model is particularly sensitive to extreme deviations from standard permissible limits, which explains why the reported values significantly exceeded the conventional ranges. This diagnostic approach aligns with the findings of Mor *et al*. (2023), who emphasised that such indices are essential for identifying high-risk zones in environments impacted by source-point pollution, such as landfills.

PCA and PCoA identified nutrients and microbial indicators as the primary drivers of water quality variation in this study. The transition of monitoring wells into the very poor category (WQI > 100) suggests that seasonal factors, particularly during the rainy season, facilitate the episodic migration of leachate into the surrounding subsurface. These seasonal patterns are consistent with those reported by Rahmanian *et al*. (2015) and Iwar *et al*. (2021), who noted that monsoonal rainfall often increases pollutant loads through runoff and infiltration. Furthermore, the mobility of contaminants such as Mn observed in the monitoring wells aligns with the findings of Nadaraja *et al*. (2017), who reported that aquifer recharge events can enhance contaminant dispersion in groundwater systems. While deeper groundwater currently remains within acceptable limits, these findings mirror the alarming threat described by Hussein *et al*. (2021) regarding the long-term migration of heavy metals and organic matter from landfills into surrounding soil and water matrices.

NBS as a strategic remediation framework given the extreme organic and microbial loading identified, NBS are recommended as a sustainable remediation strategy. Adopting the framework proposed by Mohd Nazir *et al*. (2025) (previously cited as Noor *et al*. 2025), which integrates natural processes with community engagement and cost-effectiveness, offers a transition toward the ecological rehabilitation. Previous studies, including Hale *et al*. (2023) and Vassalle *et al*. (2023), have demonstrated that NBS interventions, such as constructed wetlands and multistage bioponds, can achieve high pollutant removal efficiencies, ranging from 80%–95% for and 70%–90% for. Furthermore, as highlighted by Zalaznik *et al*. (2023), NBS not only provides technical water treatment but also offers co-benefits such as biodiversity enhancement and the transformation of landfill sites into green landscapes. This integrated approach is essential for long-term water resource management in industrialising provinces like Chachoengsao

## CONCLUSIONS

Water quality around the landfill site in Chachoengsao Province differed markedly among monitoring locations. The waste trap and monitoring ponds showed very high WQI values at various times and were classified as inferior water quality. In contrast, surface water sources, including Khlong Tha Lat and Huai Ban Song, generally remained in the good-to-moderate range, and groundwater from deep wells maintained good quality, with no heavy metals or toxic substances detected at concentrations exceeding standards. However, some monitoring wells showed episodically elevated values, which may indicate leachate leakage into groundwater, particularly during periods of heavy rainfall.

Statistical analyses indicated that the main variables influencing water quality were nutrients and microorganisms, particularly BOD_5_, NH_3_-N, TCB and FCB, which were associated with seasonal conditions and pollution sources. The results also indicated the need for preventive and restorative measures, particularly the application of NBS. Wastewater management based on natural processes, such as constructed wetlands that remove nutrients and heavy metals through filtration and uptake by aquatic plants and microorganisms, and multistage bioponds that accelerate organic matter degradation, represents a practical approach for leachate management. This research suggests that NBS can reduce BOD_5_ by 80%–95%, reduce NH_3_-N by 70%–90%, and effectively reduce microbial contamination. In addition, NBS can be cost-effective and environmentally sustainable, and can support long-term transformation of landfill areas into green spaces or environmental learning resources. Therefore, integrating NBS into Thailand’s solid waste management system is a potential strategy to enhance the sustainability of local and national water resource and environmental management.

## Figures and Tables

**FIGURE 1:**
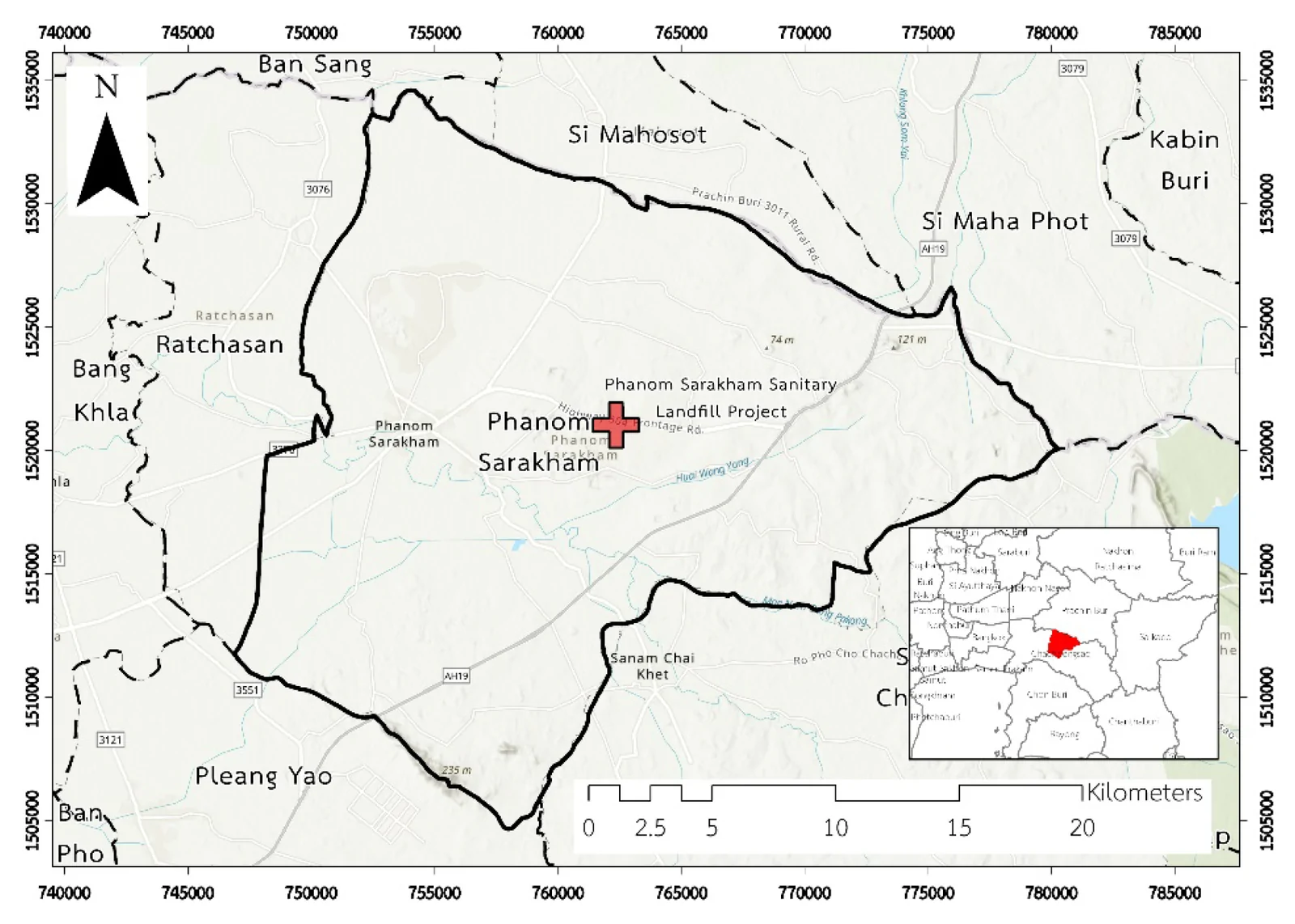
Map of research site located on the sanitary landfill site in Chachoengsao Province.

**FIGURE 2:**
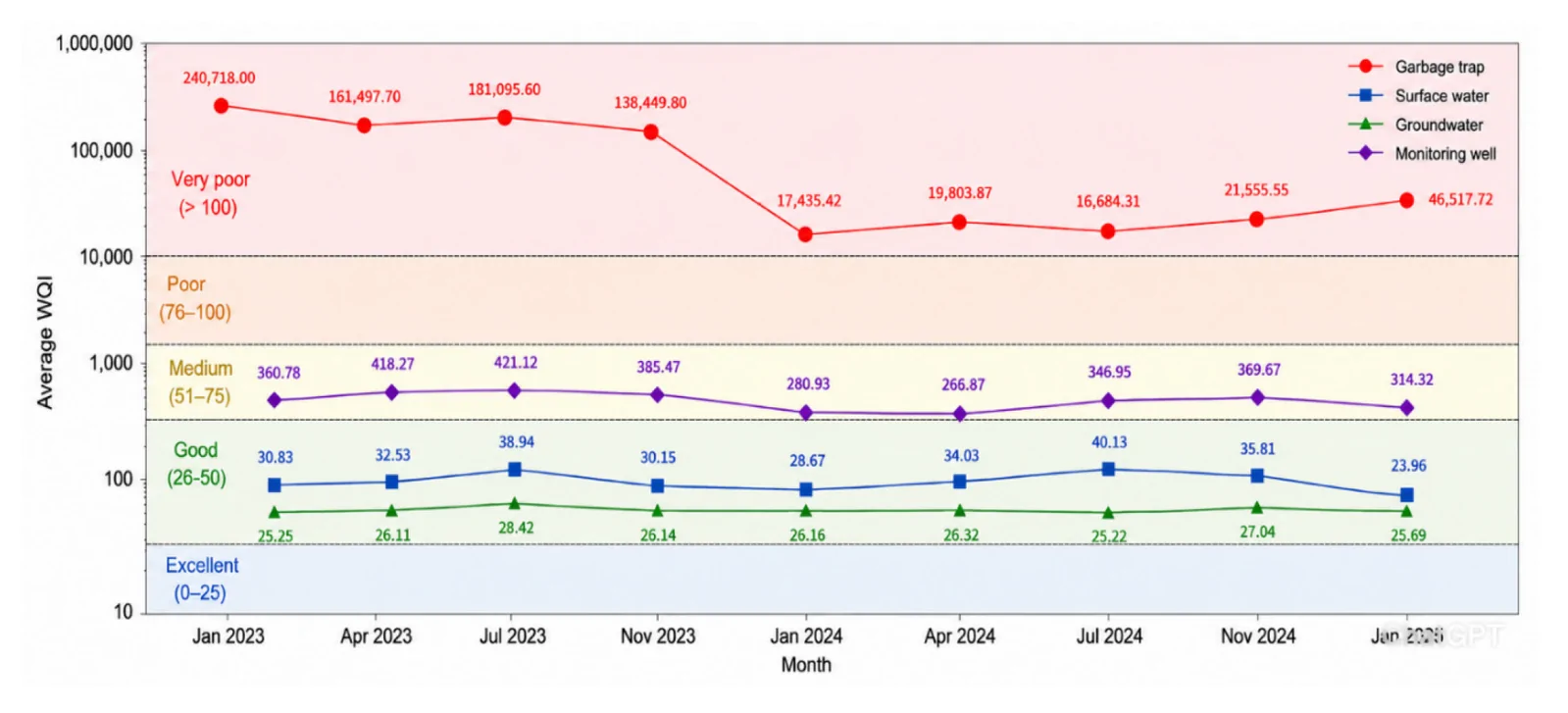
Temporal variation in the average Water Quality Index (WQI) of four water sources surrounding the landfill (garbage trap, surface water, groundwater and monitoring wells) from January 2023 to January 2025. The Y-axis is presented on a logarithmic scale to accommodate the wide range of WQI values in the study. The data indicated consistently higher WQI values in the garbage trap than in the other water sources, with progressively lower values observed in the monitoring wells, surface water and groundwater.

**FIGURE 3:**
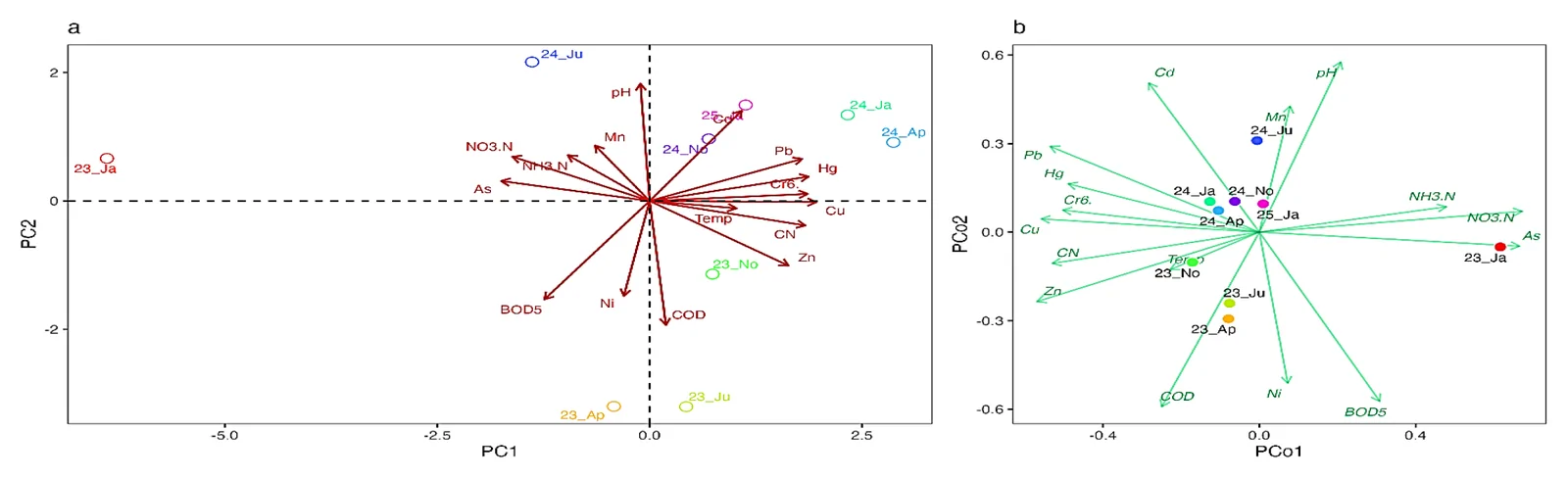
Water quality analysis at the garbage trap site: (a) PCA and (b) PCoA. Abbreviations: January 2023 (23_Ja), April 2023 (23_Ap), July 2023 (23_Ju), November 2023 (23_No), January 2024 (24_Ja), April 2024 (24_Ap), July 2024 (24_Ju), November 2024 (24_No), and January 2025 (25_Ja)

**FIGURE 4:**
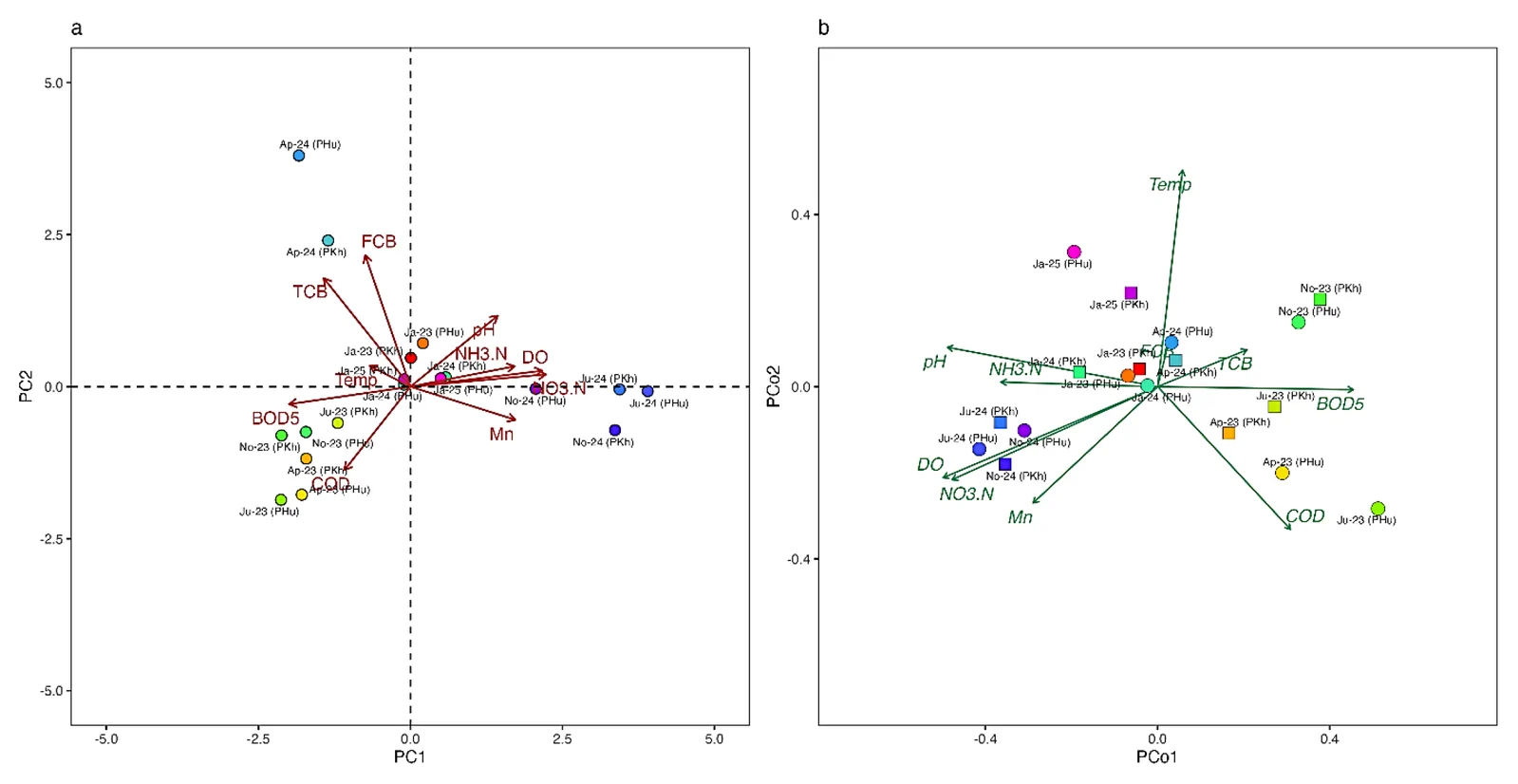
Water quality analysis at surface water sites in Khlong Tha Lat (PKh) and Huai Ban Song (PHu): (a) PCA and (b) PCoA. Abbreviations: January 2023 (23_Ja), April 2023 (23_Ap), July 2023 (23_Ju), November 2023 (23_No), January 2024 (24_Ja), April 2024 (24_Ap), July 2024 (24_Ju), November 2024 (24_No), and January 2025 (25_Ja).

**FIGURE 5:**
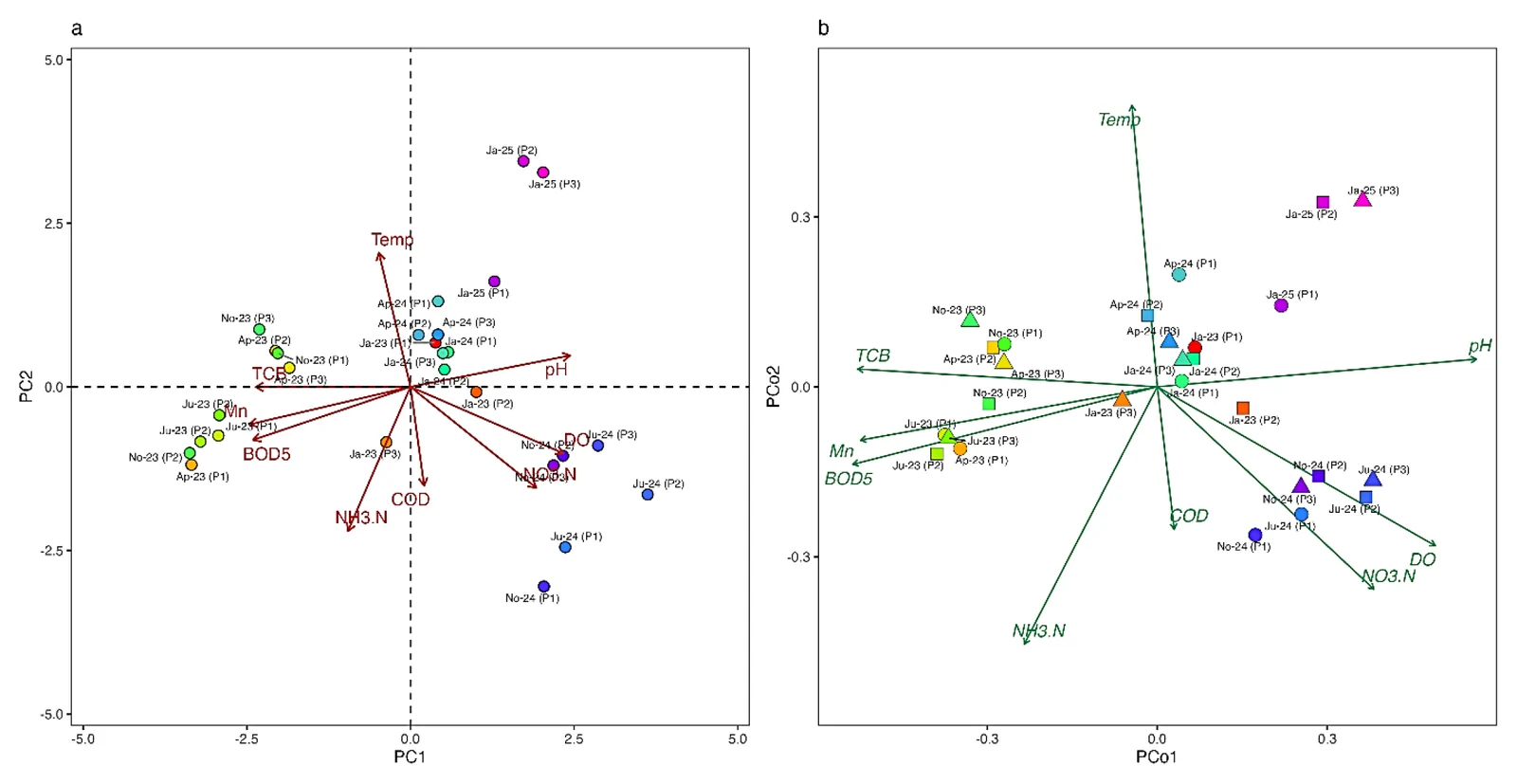
Water quality analysis at the monitoring well sites in Pond 1 (P1), Pond 2 (P2) and Pond 3 (P3): (a) PCA and (b) PCoA. Abbreviations: January 2023 (23_Ja), April 2023 (23_Ap), July 2023 (23_Ju), November 2023 (23_No), January 2024 (24_Ja), April 2024 (24_Ap), July 2024 (24_Ju), November 2024 (24_No), and January 2025 (25_Ja).

**FIGURE 6:**
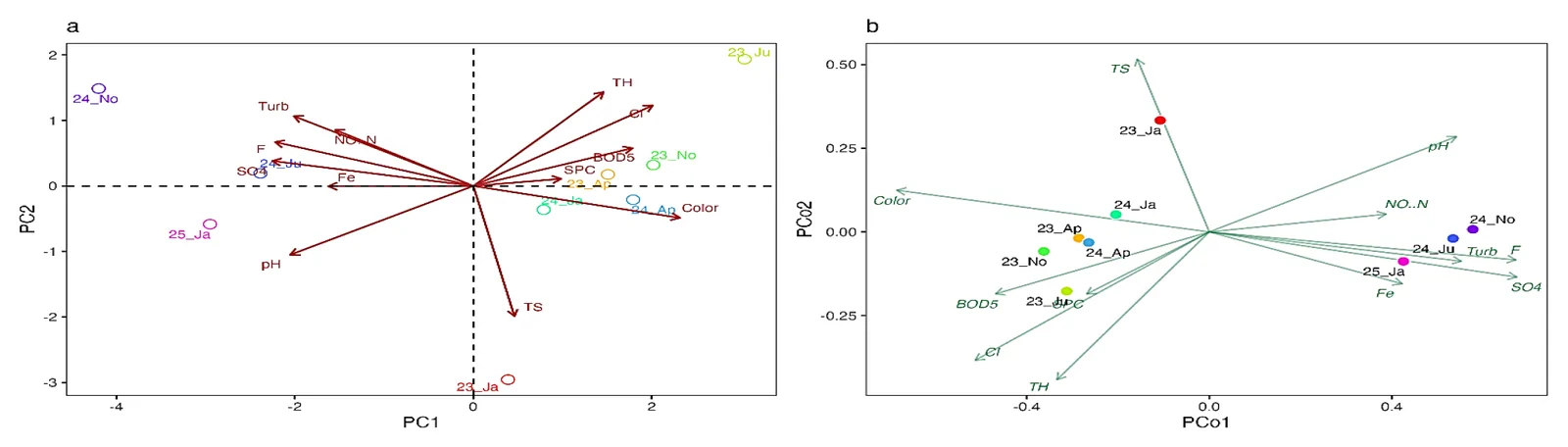
Water quality analysis at the groundwater site: (a) PCA and (b) PCoA. Abbreviations: January 2023 (23_Ja), April 2023 (23_Ap), July 2023 (23_Ju), November 2023 (23_No), January 2024 (24_Ja), April 2024 (24_Ap), July 2024 (24_Ju), November 2024 (24_No), and January 2025 (25_Ja).

**TABLE 1:** Water quality classification based on WQI classification standards (Sahu & Sikdar 2008; Mor *et al*. 2023).

WQI value	Rating of water quality	Qualification	Colour
0–25	Excellent	Clean water, excellently suitable for drinking	Blue
26–50	Good	Suitable for drinking	Green
51–75	Medium	Modestly suitable for drinking	Yellow
76–100	Poor	Unsuitable for drinking, minor treatment (purification) required before usage	Orange
> 100	Very poor	Unsuitable for drinking, appropriate treatment required before usage or seek alternative sources of supply	Red

**TABLE 2:** Average values of water quality parameters in the study area.

Parameters	Unit	Average of water quality						

		Garbage trap	Surface water	STD of surface water	Ground water	STD of ground water	Monitoring well	STD of monitoring well
pH	-	6.97 ± 1.62	7.62 ± 0.38	5.9–9.0	7.75 ± 0.41	6.5–9.2	7.49 ± 0.46	-
BOD_5_	mg/L	9,550.32 ± 9,790.76	1.07 ± 0.55	< 2.0	1.76 ± 1.57	-	6.84 ± 4.29	-
NH_3_-N	mg/L	317.12 ± 485.80	0.41 ± 0.23	< 0.5	-	-	5.19 ± 1.67	-
NO^−^_3_ -N	mg/L	36.98 ± 58.56	0.58 ± 0.64	< 5.0	0.41 ± 1.01	< 45.0	0.61 ± 0.83	-
DO	mg/L	0.00	6.88 ± 1.15	> 4.0	-	-	6.71 ± 1.05	-
TS	mg/L	-	-	-	85.75±50.33	-	-	-
TCB	MPN/100 mL	> 160,000	86.67 ± 58.23	< 20,000	< 1.1	< 2.2	86.80±21.09	-
FCB	MPN/100 mL	> 160,000	57.50±10.61	< 4,000	-	-	< 1.8	-
SPC	CFU/mL	-	-	-	36.88±41.04	< 500.0	-	-
*E.coli*	CFU/mL	-	-	-	N.D.	None	-	-
Temperature	^o^C	25.47 ± 0.63	25.32 ± 0.64	-	0.00	-	25.36 ± 0.63	-
Turbidity	NTU	-	-	-	0.09 ± 0.13	< 20.0	-	-
Mn	mg/L Mn	9.47 ± 19.42	0.12 ± 0.18	< 1.0	< 0.001	< 0.5	0.02 ± 0.01	< 0.5
COD	mg/L	15,940.17 ± 13,516.58	44.25 ± 2.87	-	< 40	-	51.09 ± 11.08	-
Colour	Hazen	-	-	-	5.00 ± 0.00	< 15.0	-	-
Total-hardness	mg/L	-	-	-	21.62 ± 22.55	< 500.0	-	-
Non-hardness	mg/L	-	-	-	N.D.	< 250.0	-	-
F^−^	mg/L	-	-	-	0.36 ± 0.12	< 1.0	-	-
As	mg/L	0.57 ± 0.73	N.D.	< 0.01	N.D.	< 0.05	N.D.	< 0.01
Cd	mg/L	0.54 ± 0.26	N.D.	< 0.005	N.D.	< 0.01	N.D.	< 0.003
Cr^6+^	mg/L	1.48 ± 0.62	N.D.	< 0.05	0	-	N.D.	< 0.05
Cu	mg/L	0.70 ± 0.21	N.D.	< 0.1	-	-	N.D.	< 1.0
Cl^−^	mg/L	-	-	< 0.005	15.40 ± 13.64	< 600.0	-	-
CN	mg/L	0.19 ± 0.06	N.D.	-	N.D.	< 0.1	N.D.	< 0.2
Fe	mg/L	-	-	-	0.04 ± 0.02	< 1.0	-	-
Pb	mg/L	0.64 ± 0.14	N.D.	< 0.05	N.D.	< 0.05	N.D.	< 0.01
Hg	mg/L	0.40 ± 0.16	N.D.	< 0.002	N.D.	< 0.001	N.D.	< 0.001
Ni	mg/L	0.46 ± 0.21	N.D.	< 0.1	-	-	N.D.	< 0.02
Se	mg/l	-	-	-	N.D.	< 0.01	-	-
SO^2−^_4_	mg/L	-	-	-	2.84 ± 0.32	< 250.0	-	-
Zn	mg/L	2.48 ± 0.18	N.D.	< 1.0	N.D.	< 15.0	N.D.	< 5.0

*Note*: N.D. = not detected

**TABLE 3:** Average of WQI across different water sources surrounding the landfill (2023–2025).

Month	Garbage trap	Surface water	Groundwater	Monitoring well
January 2023	240,718.00	30.83	25.25	360.78
April 2023	161,497.70	32.53	26.11	418.27
July 2023	181,095.60	38.94	28.42	421.12
November 2023	138,449.80	30.15	26.14	385.47
January 2024	17,435.42	28.67	26.16	280.93
April 2024	19,803.87	34.03	26.32	266.87
July 2024	16,684.31	40.13	25.22	346.95
November 2024	21,555.55	35.81	27.04	369.67
January 2025	46,517.72	23.96	25.69	314.32
